# Prevention Strategies in Obesity Management: A Systematic Review Comparing Canadian and American Guidelines for Adults

**DOI:** 10.7759/cureus.71550

**Published:** 2024-10-15

**Authors:** Adanna Ijoma, Saidat A Akanbi, Etinosa A Idemudia, Lara Aderemi, Victoria O Titus, Tricia O Okoye, Damilola A Adeyemo, Rachel A O’dare, Okelue E Okobi

**Affiliations:** 1 Anaesthesia, Red Deer Regional Hospital Centre, Red Deer, CAN; 2 Preventive Medicine, Boston University, Boston, USA; 3 Psychiatry, North Vista Hospital, Las Vegas, USA; 4 Family Medicine, Lagos University Teaching Hospital (LUTH), Lagos, NGA; 5 Internal Medicine, Healthy Choice Family Clinic, Largo, USA; 6 General Medicine, Ambrose Alli University College of Medicine, Benin City, NGA; 7 Family Medicine, Texas A&M (Agricultural and Mechanical) University, Corpus Christi, USA; 8 Nursing, South University, Savannah, USA; 9 General Medicine, Medical University of Graz, Graz, AUT; 10 Family Medicine, Medficient Health Systems, Laurel, USA; 11 Family Medicine, Lakeside Medical Practice, Belle Glade, USA; 12 Family Medicine, Larkin Community Hospital Palm Springs Campus, Miami, USA

**Keywords:** guideline, management of obesity, obesity medicine, prevention programs, teaching and learning pedagogy and education material characterization management materials learning professional development business academic writing strategic management

## Abstract

The fast-increasing obesity prevalence rates in children, youths, and adults in the last decade have made obesity prevention a global public health priority. The primary objective of this study is to evaluate the various obesity prevention strategies and guidelines implemented in the United States and Canada. Thus, for this study, a systematic review was performed on various online databases including PubMed, Scopus, Google Scholar, and MEDLINE. The decision to study the obesity prevention strategies in Canada and the United States is a result of the high prevalence rates of obesity in the two countries, alongside the numerous prevention interventions that have been executed to prevent obesity. Additionally, the systematic review used robust methodology that followed the Cochrane guidance and Preferred Reporting Items for Systematic Reviews and Meta-Analyses (PRISMA) guidelines. Only studies published between 2014 and 2024, drawn from listed databases, were included in this systematic review. The quality of the included studies was evaluated using the appraisal tool for cross-sectional studies, with the studies being rated moderate to high quality. Therefore, a total of 15 studies met the inclusion criteria and were reviewed. The findings indicate that various obesity prevention interventions have been implemented across the United States and Canada, with diverse degrees of success in obesity prevention and management. Food labeling, regular exercises, portion size regulation, school-based intervention strategies, early childhood Intervention programs, and sugar-sweetened beverage taxation were found to be effective interventions for preventing obesity in children and adults. Based on the findings, there is a need to ensure full execution of the different interventions to ensure significant reduction in obesity prevalence, as well as prevention of obesity in different populations.

## Introduction and background

Obesity is an increasingly common metabolic disease affecting individuals of all ages, from children to the elderly. At present, obesity has reached epidemic proportions in developed nations, including the United States and Canada, as well as in developing nations. The past two decades have witnessed obesity becoming the most prevalent nutritional challenge globally, obscuring infectious diseases and undernutrition as the most substantial contributors to mortality, morbidity, and ill health [1-3]. Globally, it is approximated that 2.5 billion individuals aged 18 years and above are obese, while approximately 160 million children are obese [1-5]. Obesity also remains a major risk factor for several non-communicable and chronic illnesses. In the United States, data drawn from the National Health and Nutrition Examination Survey (NHANES) have indicated that over two in every five adults (approximately 42.4%) have obesity, even as one out of every 11 adults have chronic/severe obesity [1-3]. Still, in children and adolescents, data from the NHANES have disclosed that, in the United States, one out of every five children and adolescents (approximately 19.3%) aged between two and 19 years has obesity, while two out of 19 (6.1%) children and adolescents with obesity have been acknowledged to suffer from severe obesity [1-3].

Consequently, in Canada, the obesity prevalence rate has increased over the years in both children and adults. The most recent Canadian Community Health Survey (CCHS) estimates indicate that one out of every four (23%) Canadians is obese [2-4]. Still, the data from the CCHS also indicate that, in Canadian children, obesity prevalence rates are advancing faster than in adults and that one out of every four children (26%) aged between two and 17 years in Canada is overweight [2-5]. The absolute number of obese individuals in the United States and Canada indicates the pressing public health challenge that has demonstrated no sign of improvement yet despite the execution of different interventions. The study has mainly focused on the United States and Canadian guidelines for management of obesity as a result of the high prevalence rate of obesity, alongside the effective prevention interventions that have been developed to reduce the prevalence rates. As such, studying the successful interventions and policy guidelines will assist in the development of effective interventions for prevention and management of obesity across the globe. Therefore, this study aims to evaluate and present the various interventional approaches and guidelines employed in the United States and Canada to prevent obesity in children and adults. The data and findings of this study are important as they will aid in the development of guidelines and public health policies that are effective in preventing obesity through the elimination of risk factors for the disease.

## Review

Materials and methods

A systematic review was conducted using an in-depth literature search on diverse virtual databases, including PubMed, Google Scholar, Scopus, and Web of Sciences, for peer-reviewed and published literature focusing on obesity prevention interventions and guidelines in the United States and Canada. To identify the studies, the search utilized Boolean operators with a practical combination of Medical Subject Headings (MeSH) terms, including obesity, obesity prevention/control, food labeling, nutrition policy, and exercise therapy. Further, the search strategy involved two phases and utilized the Preferred Reporting Items for Systematic Reviews and Meta-Analyses (PRISMA) guidelines for selecting and including articles for systematic reviews. The initial phase entailed performing an independent screening of the title and abstract of each retrieved article by two researchers. If there is insufficient data in the study's abstract to inform the retention or exclusion decision, the exclusion decision for the article was only arrived at after full-text screening. Nevertheless, articles with inadequate abstract data but a relevant title to the systematic review were included in the second phase, which involved full-text screening. The second phase entailed the full-text screening of the retained articles for the study inclusion and exclusion criteria. All potential disputes were solved through a third researcher tasked with making a final decision on whether a disputed article should be included or excluded, and the decision is mainly arrived at via consensus and consultations.

The search on the databases and other sources yielded 610 references. Following removing 132 duplicates, 478 unique records were retained for screening. Additionally, a total of 371 articles were removed during the initial screening of the articles' abstracts and titles as a result of their irrelevance to the study topic, as their abstracts indicated that their objectives diverged from the study, including their focus on interventions executed in countries other than the United States and Canada. This was followed by the independent assessment of 107 full-text articles determined to be relevant to the study based on the present criteria for selection. The full-text assessment led to the discarding of 92 studies for different reasons, including (i) irretrievable full text (26 articles), (ii) protocol issues (19 articles), (iii) failure to report on study limitations (10 articles), (iv) misalignment with the study objectives (12 articles), and (v) studies published in non-peer-reviewed journals (10 articles). Eventually, 15 studies satisfied the inclusion criteria, which led to their inclusion in the qualitative synthesis. The summary of the findings of the studies included in this systematic review has been presented in the Appendix, Table 1.

Study inclusion and exclusion criteria.

**Figure 1:**
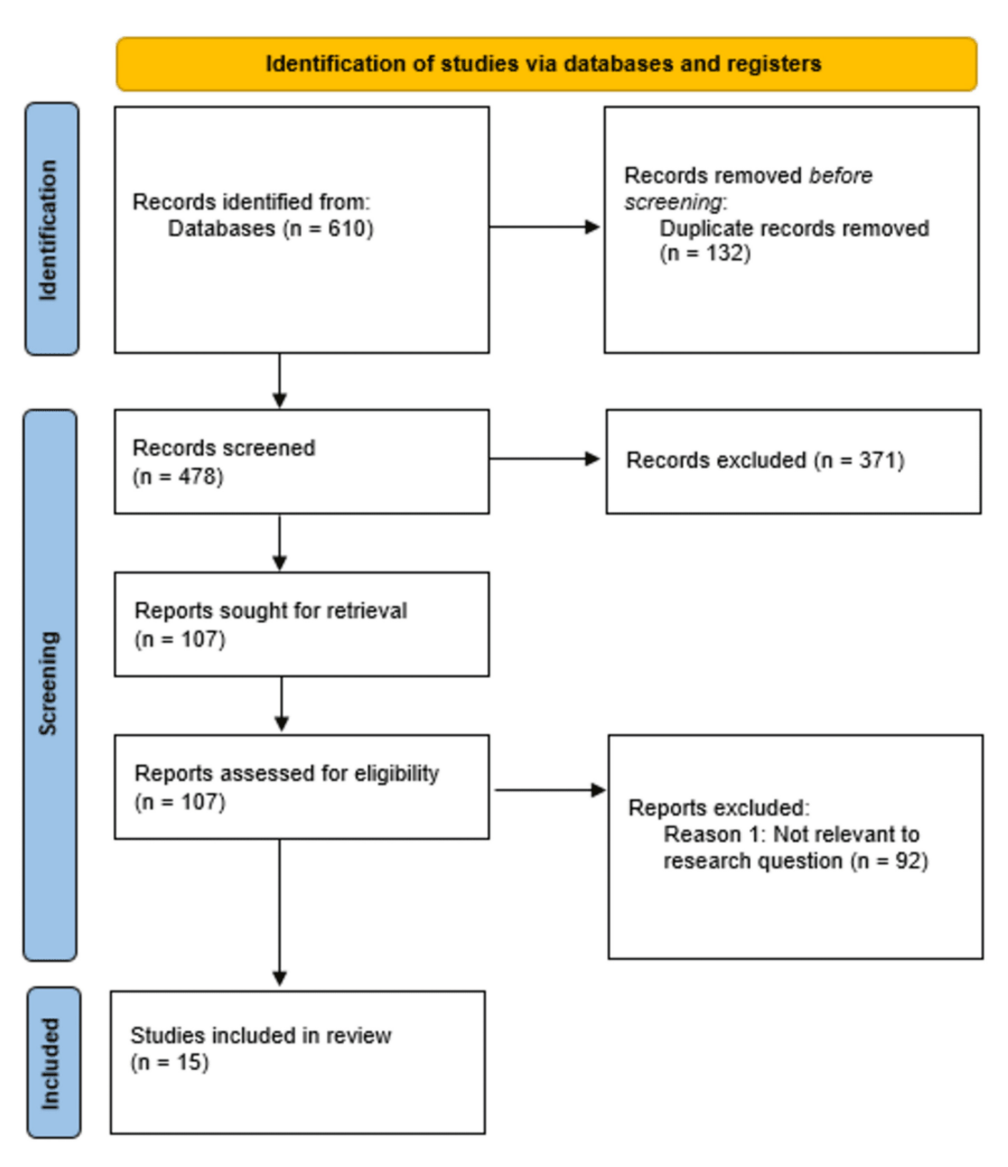
PRISMA flow diagram indicating the study selection process for the studies included in this systematic review PRISMA: Preferred Reporting Items for Systematic Reviews and Meta-Analyses.

This study's inclusion criteria included original studies, like crossover design studies, prospective cohort studies, and randomized controlled trials, focusing on obesity prevention interventions and guidelines in the USA and Canada. For this systematic review, only original studies were included, as they offered primary data that was up-to-date and pertinent to the study period and findings. Moreover, only English-language literature and guidelines published in the last 10 years were included. The studies excluded were various types of sponsored clinical trials, editorials, opinion pieces, and narrative reviews. Following the evaluation of titles and abstracts, 371 articles were excluded. This was followed by extracting information from the eligible studies based on the general study attributes, including the authors' names, study year, publication year, and method of sampling employed, and the study population characteristics, including the gender, age, race, and sample size, and the type and duration of intervention, and the main findings. A detailed overview of the literature searches and selection process for this study using PRISMA is presented in Figure 1 below.

Data collection process

Data extraction from the included literature was conducted under the author's supervision. Potential discrepancies and disputes were solved through discussions and consultations with the third reviewer, even as the extraction of data on the study author, publication year, study location, screening tool used, sample size, and rate of response was done independently by the author using a standard format for data extraction. The included studies' quality assessment was conducted using the Joanna Briggs Institute quality assessment tool [3]. Therefore, every publication scored using a frequency scale with yes, no, unclear, and inapplicable responses. Each study’s overall quality score was computed based on the total positive scores received.

Data analysis

The study attributes, including the research design employed, sample size, year of publication, and location of the study, were utilized in evaluating potentially heterogeneous sources. For each statistical analysis conducted about statistical significance, the P-value was maintained at 0.05.

Review of literature and discussion

The causes of obesity remain multifactorial and intricate. Within environmental, genetic, and social factors, obesity results from the long-term balance of positive energy, which entails the interaction between energy consumption and energy expenditure. The fast increase in the prevalence rates of obesity observed in the last two decades can be attributed to environmental and cultural influences as opposed to genetic factors. With the gradual improvements in living standards in both developed and developing nations, sedentary lifestyles alongside over-nutrition have supplanted regular physical activities and physical labor, leading to positive energy balance and obesity/overweightness [4]. Significant advancements in physical activities/exercises, dietary, bariatric surgical, behavioral, and pharmacological interventions have resulted in effective long-term obesity management. Nonetheless, lifestyle-based interventions have remained the cornerstone of obesity management and treatment, even though adherence remains poor alongside modest, long-term success, owing to the substantial barriers attributable to the affected persons and healthcare professionals tasked with the provision of treatment. Bariatric surgery and pharmacotherapy are important adjuncts for the enhancement of the health outcomes of obese/overweight persons, even though, for several reasons, such treatment modalities have not been broadly adopted. The health promotion and prevention interventions for obesity mainly aim to maintain healthy weights and avoid comorbid diseases. The classification by the World Health Organization (WHO) has distinguished between the selective and general classification [4]. Thus, prevention intervention programs have been categorized as behavior-oriented and individual-based interventions or environment or community-based intervention programs as context-associated interventions [4]. The latter category supports various health-pertinent decision-making, even as environmental factors, including residential neighborhoods, positively influence weight status and health-associated behaviors [5]. Existing prevention strategies mainly focus on behavioral interventions, even as existing gaps concerning obesity prevention are found in the environment and community-based interventions.

Early childhood intervention programs in obesity prevention

Of the included studies in this systematic review, a total of three studies reviewed various early childhood intervention programs for preventing obesity [5-12]. The intervention strategies reviewed in the studies include physical activities, screen time restriction, and healthy nutrition and dietary habits. In this regard, it is noteworthy that, globally, childhood obesity affects nearly 38.3 million, or 5.6% of children below the age of five years. The longer or more a child remains obese or overweight, the more likely he/she is to become obese as an adult with each of the involved contingent morbidity. Thus, early care and education (ECE) contexts have been reported to represent the most significant point of intervention for the prevention of childhood obesity. Interventions such as active engagement in physical activity, restriction of screen time, and maintenance of nutritious diets have been linked to healthy weights in children [5-7]. Although the evidence relating physical activity to adiposity during early years remains limited, recent studies have disclosed a relationship between physical activity and healthier cardiometabolic profiles, enhanced cognitive and motor development, and improved skeletal health [5,8]. In this regard, the Canadian 24-Hour Movement Guidelines for the Early Years has proposed that toddlers and preschoolers should be accorded a minimum of 180 minutes of everyday physical activity at a different intensity and that, of the 180 minutes, preschoolers should be accorded 60 minutes of moderate and vigorous-intensity physical activity (MVPA) [6,9]. Consequently, the recommendations for screen time restrictions are mainly based on the findings of studies that disclosed that there were significant correlations between excessive screen time and adiposity, even as the possible mechanisms underpinning the correlation include increment in sedentary times, disruption of the normal sleep patterns for the children, and increased exposure to unhealthy foods and beverages advertisements [6,10,11]. Recent Canadian data collected between 2009 and 2015 indicates that while 61% of the nation’s preschoolers meet the recommended physical activity guidelines, fewer than a quarter adhere to the recommended limits for screen time [12,13].

To address obesity and overweight in children, Canada's Food Guide promotes healthy nutrition and dietary habits. It encourages increased consumption of vegetables, fruits, lean proteins, whole grains, and water as the preferred drink. Additionally, it advises limiting processed foods high in saturated fats, sodium, and sugar [13,14]. Even though the report on the assessment of adherence to the new Food Guide for Canada (updated in 2019) has not been published, the studies on the earlier versions have disclosed that several younger children have not met the recommendations [14]. However, a recent study approximated that children aged between two and eight years old have a score of 65 points out of the possible 100 points concerning the Canadian adoption of the Healthy Eating Index (which is a measure used in the evaluation of the dietary quality in which the score of 100 shows the highest diet quality) [14].

Taxation of sugar-sweetened beverages 

In this systematic review, three studies focused on taxing sugar-sweetened beverages as an obesity management and prevention strategy [15-22]. The included studies have mainly focused on policy interventions, including taxation policies. Thus, regarding sugar-sweetened beverages taxation as a prevention strategy in obesity management, it is noteworthy that most existing policies have focused on addressing behavioral interventions for preventing obesity. However, recently, the policy on taxation of sugar-sweetened beverages as a prevention intervention for obesity has gained traction across the globe. Whereas physical activity and public nutrition education campaigns have remained the preferred intervention alternatives, more recent fiscal policies have become politically salient alternatives [15], principally sugar-sweetened beverages (SSB) taxation. The World Health Organization's (WHO) endorsement of SSB taxation policies in 2016 has invigorated the extent of its consideration by various nations across the globe [16]. In this regard, the WHO maintains that imposing taxes on SSB offers a potential approach to increasing the prices of various SSB products, which, in turn, considerably reduces their consumption [17]. The findings of a recent review study have indicated that, generally, an increase in taxes tends to decrease the consumption of the affected taxed beverages, including SSB beverages, by adults, even though not for every kind of beverage or even all consumer groups [18].

Additionally, studies have shown a positive impact of the SSB taxations, including a decrement in sales, purchases, and consumption in instances where the increased taxes on SSB products increased their prices, especially in upper-income nations such as the United States and Canada [19,20]. In this regard, the findings of this systematic review indicate that SSB taxation is likely to prove a productive financial policy for decreasing the sale, purchase, and consumption of various SSB products while simultaneously reducing the prevalence rate of obesity and overweight. The impact of sugar-sweetened beverages (SSBs) would be greater in cases where both their consumption is higher and where specific measures are in place to track beverage volumes and include all SSBs. The study's support for fiscal policy interventions mainly stems from the observation that product price modification can considerably alter consumption [21]. Still, through the increment of SSB prices, a considerable reduction in the price gap with beverages considered healthy, including milk and water, is prone to occur, promoting the sale, purchase, and consumption of healthy beverage alternatives [22]. A simple food price manipulation is likely to alter consumption patterns in a way that might eventually reduce individual development of diet-associated diseases [16].

Notably, the City of Berkeley, California, implemented a $ 0.01 excise tax for each fluid ounce on SSB products in early 2015, thereby becoming the initial jurisdiction in the United States to execute an excise tax applicable to both the manufacturers and re-sellers of SSB products by volume and weight [23]. Following the implementation, pre-and post-sales SSB consumption rates were compared with the neighboring cities of San Francisco and Oakland, California. Data was collected through the beverage frequency questionnaire eight months before voting for the excise tax and four months after its implementation. The researchers observed a significant reduction in the SSB consumption rate in Berkeley at 21%, compared to the moderate increment of 4% in the consumption rate of SSB in San Francisco and Oakland [23]. Still, the city of Berkeley registered a significant increment of 61% in water consumption, even as the neighboring cities also recorded a 19% increase in water consumption [23]. Further, 12 months after the excise tax on SSBs in Berkeley, the effects on prices, store revenues, sales, consumer expenditures, and beverage consumption rates were reviewed [21]. It was reported that the SSB sales in the city reduced by approximately 9.6%, even as a 6.9% increment in consumption was noted in various non-Berkeley stores [21]. Still, it was disclosed that the sale of non-taxed beverages had increased by 3.5% in Berkeley, compared to an increase of 0.5% reported in non-Berkeley outlets, mainly driven by water sales.

Dietary guidelines as a prevention strategy in obesity management

Of the included and reviewed articles in this systematic review, three studies have focused on dietary guidelines as a prevention strategy in obesity management [24-36]. The included studies have mainly focused on the reducing energy density, dietary, and consumption patterns. Therefore, concerning the dietary guidelines strategy in obesity management, it can be noted that the recent surges in obesity rates are mainly driven by food choices and preferences and the eating behaviors that promote excessive energy intake. Thus, several dietary patterns, including food and macronutrient-based diets, have been considered interventions that can result in considerable weight loss. Therefore, a major weight management strategy and intervention applicable across dietary patterns entails the reduction of energy density [24]. Various clinical trials have reported that reducing energy density is effective for weight loss and maintenance. The present weight management recommendations have emphasized the significance of healthy dietary patterns, which include an array of nutrient-dense foods, restricted portions of energy-dense foods, and a general reduction in energy density [25]. Similar weight loss can be attained through dietary patterns restricting energy intake or consumption with the amount of energy expended.

Several dietary patterns that reduce energy intake with energy expenditure lead to similar weight loss. A unifying factor for weight loss across dietary patterns is energy density. Reducing a diet’s energy density allows individuals to consume satisfying amounts of food for fewer calories. For instance, an evidence-based report developed by the American College of Cardiology and the American Heart Association Task Force about the Practice Guidelines and The Obesity Society have indicated their support for some energy-limited dietary interventions for attainment of weight loss by concentrating on macronutrients, which include lower-carbohydrate, low-fat, moderate-to-higher protein, and diets that are macronutrient-targeted [26]. Even as dietary interventions are considered effective, several studies (reviews) have disclosed that focusing on a specified macronutrient to attain the desired weight loss is unnecessary, given that diverse macronutrient proposals have resulted in comparable clinically substantial weight loss after six months, one year, and two years [27,28].

Moreover, the recent dietary guidelines have stressed the significance of considering consumption patterns and whole diets instead of the reductionist approaches focusing on single nutrients and foods [29]. For instance, the 2015 Dietary Advisory Committee has recommended that obese and overweight persons achieve the desired weight loss through healthy eating and dietary patterns [24]. Notable examples of the proposed dietary patterns include the Healthy US-Style Eating Pattern, which is representative of the Dietary Approaches to Stop Hypertension (DASH) diets, and the Healthy Mediterranean-Style Eating Pattern [25]. In this regard, the DASH eating program has recommended reductions in the consumption of less healthy fats and maintaining a total fat intake of below 25% of the total energy of the diet, in addition to increasing the percentage of low-energy-dense food, including fruits and vegetables (from nine to 12 servings every day) alongside low-fat dairy products from two to three servings every day [30]. As an eating pattern, the DASH diet is considered to be low in energy density. It enables individuals to consume less energy without decreasing their food weight [31]. Consequently, the Mediterranean diet has also stressed consuming low-energy-dense vegetables, fruits, dairy foods, seafood, and legumes [32]. Nonetheless, higher fat amounts, approximately 30% to 40% of the overall energy, particularly from olive oil, have been recommended alongside the Mediterranean diets [32]. Regardless of the above level of recommended healthy fats, a higher proportion of fruits and vegetables that have been included in these eating patterns are capable of aiding in maintaining and ensuring an overall diet that is comparatively low in energy density [33]. Reducing the dietary energy density is also prone to aid obese and overweight persons in maintaining and sustaining their weight loss efforts [34,35]. For example, a weight loss program conducted in a clinical setting, which promoted the consumption of low-energy-dense diets, disclosed that persons who maintained their weight loss after two years reported consuming lower-energy-dense diets compared to the participants who reported regaining 5% and above of their body weights [36]. In an additional study, the instructions on reducing dietary energy density for obese and overweight participants resulted in sustained weight loss three years following the commencement of the intervention [24].

School-based obesity prevention programs

Of the included studies in this systematic review, three have highlighted the effects of various school-based obesity prevention programs and interventions [37-40]. Among the notable school-based obesity prevention interventions reviewed in the included studies are nutrition education, physical activity promotion, and behavioral and lifestyle changes. As such, special focus has been placed on elementary schools, owing to their acknowledgment as the ideal setting for the prevention of childhood obesity, given that the schools offer several opportunities for the execution of interventions that include nutrition education, physical activity promotion, and the decrease of sedentary behavior and lifestyles via policies, practices, and supportive environments [37]. Moreover, involving teachers in obesity prevention programs is highly effective. Training teachers by healthcare experts to deliver obesity prevention interventions, coordinate school-based activities, and promote healthy eating behaviors during school hours has shown promising results. Rather than relying solely on healthcare professionals to implement these interventions, empowering teachers offers a practical and sustainable approach. This could be further enhanced by using visual tools like Venn diagrams in public places, such as schools, where parents and children can see them, encouraging subconscious self-advice and awareness [38]. Thus, in-class materials, including manuals, posters, and workbooks, can be used to support and facilitate the intervention programs.

Further, active teacher involvement as role models in the targeted energy balance-related behaviors (EBRBs) has been acknowledged as a productive approach [39]. Several school policy-based strategies, including free fruits, increased access to clean drinking water, healthy cafeteria food alternatives, and removal of vending machines, are increasingly effective interventions for obesity prevention in school-going children [37,38]. The development of the wellness councils by the school staff, along with the written wellness policies, has also been found to be important drivers of the effectiveness of the intervention [40]. In particular, the promotion of physical activity during recess, the development of active playgrounds, and the non-competitive and gratifying activities that promote the participation of the entire class during physical education lessons, as well as the provision of extra hours for physical activity, have been demonstrated to be effective interventional strategies [38].

Portion size regulation as a prevention strategy in obesity management

Three studies in this systematic review evaluated the effects of portion size regulation as a prevention strategy and intervention in obesity management. The studies have mainly focused on how the size of food portions consumed affects obesity and obesity prevention in relation to energy intake. In this regard, portion size is considered a major environmental driver of energy consumption, as bigger foods have been acknowledged to elevate the risk of weight gain and obesity. Several studies, propped up by data drawn from free-living contexts, have shown that portion size has significant and proportionate effects on the quantity of food eaten. Importantly, overeating bouts linked to bigger portion sizes are always sustained without being followed by compensatory reductions in energy consumed. The effects of portion size on energy consumption are evident across different types of foods and beverages, particularly in calorie-dense food options [41]. Thus, individuals often overeat when offered bigger portions, irrespective of age, gender, body mass index, and socio-economic status [42]. The increased availability of big portion sizes and the value-size pricing have substantially changed the views on apt quantities of food. Even as there is no definitive direct correlation between portion size and obesity, moderation of portion sizes, especially in calorie-dense foods, is a key recommendation and intervention for weight management. The increase in portion sizes has been marked by a consequent increment in obesity rates in the United States and Canada, indicating the existence of potential correlations. Moreover, various studies conducted in the United States in the last three decades have indicated an increase in energy consumption in children and adults, and this has been attributed to an increase in frequent eating and bigger portions [33,43].

Additionally, several studies have indicated that offering individuals bigger food portions results in significant and sustained increments in energy intake [44,45]. The findings of the studies indicate that access to larger portions may override energy balance regulation and have obstinate effects that might, in turn, result in obesity development. In this regard, the Dietary Guidelines Advisory Committee study conducted in 2010 disclosed a sturdy positive correlation between portion size consumed and body weight [46]. The study's findings underscore the significance of portion size regulation in limiting the amount of energy consumed and preventing obesity development. Similarly, in the Research to Practice Report 2006, the Centers for Disease Control and Prevention (CDC) acknowledged that the portion size of food consumed could influence weight management [36]. Even though countering the effects of larger portion sizes is seen as simply consuming less of all things, the intervention may prove difficult for individuals to adopt and sustain in the long term [47]. However, to attain and maintain a healthy weight, it is recommended that one should substitute fruits and vegetables, along with other nutrient-rich and low-energy-dense foods, for higher-energy-density foods, as this will enable one to not only eat more but get the given amount of calories necessary to maintain a healthy weight and prevent obesity [48].

Food labeling requirements and their role in obesity prevention

Of the 15 studies included in this systematic review, a total of eight studies have reviewed the effects of food labeling requirements concerning obesity prevention. The included studies have mainly focused on the role of food and nutrition labeling in preventing obesity, with a special focus on nutrition labeling as a means of creating awareness of the food contents. Thus, it is noteworthy that making healthier dietary decisions requires the development of food environments that promote the consumption of healthy diets. Within the food environment, nutritional labeling serves two key purposes: consumer health protection and ascertaining fair trade practices by food manufacturers [49]. Thus, nutrition labeling notifies the consumers of the food product’s nutritional attributes to assist in purchasing and consumption decision-making and prevent false, deceptive, and misleading labeling. Packaged food labeling is the principal communication between the food manufacturer and the buyer/consumer [50]. As an intervention, food labeling can aid in rebalancing the food retail milieu that is presently skewed toward diets and foods, undermining healthy diets by providing information on foods' quality and nutritional attributes. As an intervention, the food labeling requirement assists consumers in determining and making decisions on the right food to eat, and this helps ensure that individuals purchase and consume foods with the correct amount of energy needed to maintain healthy weights and prevent obesity [51].

Further, extant evidence regarding the effects of nutrition labeling is derived from studies that evaluated the nutrition labeling systems’ performance [52], as well as the effect of specific labeling designs and various content aspects about behavioral outcomes, including choice, comprehension, awareness, and dietary consumption [53,54], as opposed to the evaluation of the various nutrition labeling policies in general. Further, Cecchini and Warin (2016) conducted a study to evaluate the effects of food labeling on both food preference and calorie intake in the US, Australia, the UK, Canada, and France [55]. The data on the overall proportion of consumers choosing and consuming healthier food products and changing their calorie consumption following the execution of food labeling policy interventions was extracted. The findings indicated that food labeling increased the percentage of individuals opting for healthier alternatives by approximately 17.95%, from 11.24% to 24.66% [55].

Still, explicit “black stop signs” placed on food packaging have been acknowledged as highly effective in assisting consumers in recognizing higher sugar, fat, and salt content, which aid in preventing obesity and other chronic conditions [56]. Such foolish labeling discourages the long-term consumption of unhealthy products while reducing unhealthy and poor dietary habits. A notable example of the effective execution of food labeling is Chile. Being a key consumer of high-sugar content beverages, Chile introduced and executed the initial compulsory front-package labeling policy in 2016 as part of the wider set of policies to prevent obesity [56]. The measures introduced by the policy included restricting and banning the marketing and sale of unhealthy drinks in schools to children. The outcomes of the policy include the observation that, in 18 months, the sale of high-energy-dense and high-sugar and fat foods and beverages reduced by approximately 25% [56]. The success story of Chile has inspired policymakers across the globe to consider comparable food labeling interventions, and researchers have acknowledged the intervention as being among the best and most appropriate methods for preventing obesity.

Exercise prescription in primary care as a prevention strategy for obesity

Of the 15 studies included in this systematic review, a total of eight studies have assessed exercise prescription in primary care as a prevention intervention and strategy for obesity. The included studies have mainly focused on exercise interventions, including aspects such as duration of exercise interventions, regularity of exercises, and types of exercises, including resistance training and physical exercises. Thus, it has been acknowledged that, in managing and treating obesity, lifestyle-based interventions and exercise should be a principal element of management and medical care [57]. Thus, the WHO guidelines recommend a minimum of 150 to 300 minutes of moderately intense physical exercises or 75 to 150 minutes of vigorous aerobic physical activity per week to attain healthy weight in adults [58]. Various common exercise prescriptions have been developed and have proven successful in weight management in obese persons [59]. For the attainment of healthy weights and effective management of obesity, the proposed important exercise prescription parameters include long (duration), often (frequency), and hard (intensity), which an individual is required to work out to attain the optimal outcomes for the body composition in terms of fat/weight loss and preservation of muscles, as well as physical functioning. Regardless of these recommendations, the effects of regular exercise on health are still underestimated [57].

Several studies have documented statistically significant weight loss, with the weight loss ranging from 1 kg to 7.5 kg, as well as reductions in the body mass index (BMI), with a reduction range of 0.3 kg to 2.34 kg/m^2^, following successful completion of various exercise interventions [60,61]. Additional studies have reported statistically significant reductions in body fat mass, as assessed using dual X-ray absorptiometry, magnetic resonance imaging (MRI), and bioelectrical impedance analysis [60-62]. The observed changes reported by the studies included a reduction in visceral adiposity (between 0.2 kg and 1.1 kg), a reduction in overall fat mass (between 5.4 kg and 7 kg), as well as a reduction in body fat percentage (between 1% and 3.97%) [59,60]. Further, high-intensity regular exercise resulted in the highest amount of body mass/fat reduction and the greatest weight loss, regardless of the mode of exercise undertaken [60-62].

Still, at present, a larger proportion of studies have reported that exercises are increasingly effective in the prevention of obesity and management of healthy weights, with various weight loss programs combining exercises and caloric restrictions being recommended owing to their aptitude to maximize the net caloric deficit even as it reduces the fat-free mass loss [61]. Resistance training with regular aerobic exercise is prone to improve the quality and quantity of muscles, thereby, health advantages independent of weight loss [62]. Further, accumulating essential exercise and lifestyle physical activities in spasmodic bouts, as opposed to single long unbroken bits, helps improve adherence and successful weight loss and weight maintenance regimens [60-62].

## Conclusions

In conclusion, the obesity epidemic’s expansion concerning affecting different age groups, ethnic/racial groups, and socioeconomic strata, as well as communities in the United States, Canada, and other nations, has resulted in the paradigm shift toward the adoption of public health interventions and policies capable of reaching different populations and communities. Although the various interventions for obesity prevention and management have been aptly supported by diverse degrees and evidence following their implementation, there is an urgent need to ensure the full execution of these interventions to attain the desired level of reduction in obesity prevalence rates. The study findings have indicated that most studies and guidelines recommend the consumption of more vegetables, whole grains, lean proteins, fruits, and water, while reducing the intake of processed foods with high amounts of sodium, saturated fats, and sugar, as one of the best means for preventing obesity. Further, the study has disclosed that SSB taxation as an intervention reduces the consumption of sugar while lowering obesity rates, even as weight loss has been linked to the reduction of the energy density of diets consumed, mainly through the consumption of low-calorie foods. Further, the study has found that to prevent obesity in children, policy interventions should encourage the development of active playgrounds, increase physical activity time, and promote non-competitive activities. In this regard, additional studies are recommended to evaluate and identify the best means of implementing the interventions to reduce obesity rates significantly. Moreover, there is a need to perceive the obesity prevention interventions within the broader health context, in addition to encompassing the long-term, multi-generational objectives, along with obesity prevention policies requirement for application within the wider range of contexts to realize long-term shift toward better health and healthy weights.

## Appendices

**Table 1:** Description

Author	Publication year	Study title	Summary of findings
Rhodes et al. [4]	2020	Development of a consensus statement on the role of the family in the physical activity, sedentary, and sleep behaviors of children and youth.	The study stressed the significance of the family in influencing sleep patterns, physical activity, and sedentary behavior of their children and youths. The findings highlight how family settings and behaviors may support or hamper the children’s healthy lifestyle behaviors, encouraging active parental participation to stimulate healthier outcomes.
Reyna-Vargas et al. [5]	2022	Longitudinal associations between sleep habits, screen time, and overweight obesity in preschool children.	The study assessed the correlations between sleep habits, screen time, and obesity in children. Poor sleep habits and increased screen time were found to be substantial risk factors for the development of obesity in children, thereby stressing the requirement for early interventions to tackle such behaviors.
Brassard et al. [11]	2022	Development of the Healthy Eating Food Index (HEFI)-2019 measuring adherence to Canada’s Food Guide.	The study focused on the Healthy Eating Food Index (HEFI)-2019, used in the measurement of Canada's Food Guidelines adherence. The index tracks the dietary patterns and assesses how closely Canadians adhere to the national nutritional guidelines, thereby serving as an important tool for public health monitoring.
Restrepo and Cantor [17]	2020	The effects of soda taxes on adolescent sugar intake and blood sugar.	The study explored the various effects of soda taxes with regard to adolescent sugar consumption and blood sugar levels. The findings indicated that soda taxes were effective in reducing sugar intake in adolescents, indicating the potential for comparable policies to advance health outcomes linked to excessive sugar consumption.
Nakamura et al. [18]	2018	Evaluating the 2014 sugar-sweetened beverage tax in Chile: an observational study in urban areas.	The study evaluated the effects of sugar-sweetened beverage (SSB) tax in Chile and disclosed that the tax was effective in reducing SSB consumption. The study findings support the effectiveness of taxation as a public health intervention for reducing the consumption of unhealthy sugary drinks.
Falbe et al. [21]	2016	Impact of the Berkeley excise tax on sugar-sweetened beverage consumption.	The study disclosed that excise tax on SSBs was found to significantly reduce SSB consumption in the community, demonstrating the effectiveness of fiscal policies in decreasing the consumption of sugary drinks and promoting healthier dietary behaviors.
Lei et al. [25]	2022	Effects of low-carbohydrate diets versus low-fat diets on metabolic risk factors in overweight and obese adults: a meta-analysis of randomized controlled trials.	The study examined how low-carbohydrate and low-fat diets impacted metabolic risk factors in overweight and obese adults through a meta-analysis. The study disclosed that diets low in carbohydrates were more effective than diets low in fat in enhancing metabolic risk factors like blood lipid levels and weight loss.
Al Aamri et al. [26]	2022	The effect of low-carbohydrate ketogenic diet in the management of obesity compared with low caloric, low-fat diet.	The study compared the efficiency of low-carbohydrate ketogenic diets with low-fat, low-calorie diets in the management of obesity. The ketogenic diet was found to be more effective in reducing body weight and improving metabolic markers in obese individuals.
Hall et al. [33]	2022	The energy balance model of obesity: beyond calories in, calories out.	The study assessed the conventional "calories in, calories out" model of obesity and proposed the energy balance model. The findings indicate that obesity is mostly influenced by multifaceted factors beyond calorie consumption, such as hormonal, environmental, and metabolic influences.
Nally et al. [35]	2021	The effectiveness of school-based interventions on obesity-related behaviors in primary school children: a systematic review and meta-analysis of randomized controlled trials.	The study evaluated school-based interventions targeting obesity-related behaviors in school-going children. The findings have indicated that the interventions, particularly those that promote physical activity and healthy eating habits, were effectual in reducing obesity in children.
Buru et al. [38]	2020	The efficacy of school-based interventions in preventing adolescent obesity in Australia.	The study assessed the effectiveness of school-based interventions in prevention of adolescent obesity. The findings indicated that interventions that integrated physical activity, behavioral changes, and nutrition education were effective in reducing obesity rates in adolescents.
Lambrinou et al. [37]	2020	Effective strategies for childhood obesity prevention via school based, family involved interventions: a critical review for the development of the Feel4Diabetes-study school based component.	The study reviewed the effectiveness of strategies for childhood obesity prevention through school-based, family-involved interventions. It emphasizes the importance of involving families in school-based programs to enhance the success of childhood obesity prevention initiatives.
Veit et al. [42]	2020	Health, pleasure, and fullness: changing mindset affects brain responses and portion size selection in adults with overweight and obesity.	The study explored how changing one’s mindset affects the brain’s responses to food stimuli and portion size selection, especially in adults who are obese. The findings have disclosed that promotion of mindsets focused on health, fullness and pleasure may positively influence eating habits and portion control.
Ares et al. [50]	2018	Comparative performance of three interpretative front-of-pack nutrition labelling schemes: insights for policy-making.	The study evaluated three front-of-pack nutrition labeling schemes and disclosed that interpretative labeling, including nutritional labels and warning labels, was highly effective in assisting consumers in making healthier food choices.
Dvorák et al. [55]	2021	The role of individualized exercise prescription in obesity management: case study.	The study highlights the importance of individualized exercise prescriptions in the management of obesity. The findings have demonstrated that personalized exercise plans, customized to the precise needs of the affected persons, might be highly effective in promoting weight loss and enhancing overall health in obese individuals.
